# Clinical Utility of Cariprazine in the Management of Acute Schizophrenia: Current Evidence and Practical Recommendations

**DOI:** 10.62641/aep.v54i4.2145

**Published:** 2026-08-15

**Authors:** José Manuel Montes-Rodríguez, Francisco Collazos-Sánchez, José Martínez-Raga, José de Blas-Soto, Moisés Bolivar-Perálvarez, Luis Gutiérrez-Rojas

**Affiliations:** ^1^Department of Psychiatry, Hospital Universitario Ramón y Cajal, IRYCIS, 28034 Madrid, Spain; ^2^Mental Health Area of Hospital Fundación Hospitalarias Barcelona (Sant Rafael), 08035 Barcelona, Spain; ^3^Department of Psychiatry, Hospital Universitario Doctor Peset, 46017 Valencia, Spain; ^4^Department of Medicine, School of Medicine, University of Valencia, 46017 Valencia, Spain; ^5^Psychiatric Hospitalisation Unit, Hospital Universitario de Navarra, 31008 Pamplona, Navarra, Spain; ^6^Department of Psychiatry, Hospital Universitario de Badajoz, 06080 Badajoz, Spain; ^7^Department of Psychiatry, Hospital Universitario Clínico San Cecilio, 18007 Granada, Spain

**Keywords:** schizophrenia, treatment, negative symptoms, cognitive impairment, adherence, dual diagnosis, cariprazine

## Abstract

**Background:**

The management of schizophrenia in acute care units presents significant challenges, particularly in addressing negative symptoms and functional impairment. Cariprazine, a dopamine D3/D2 receptor partial agonist, offers a unique pharmacological profile in this setting. This review aims to provide an expert consensus on the clinical utility of cariprazine in acute settings and establish practical recommendations for its use.

**Methods:**

A comprehensive literature review was conducted across specialized databases, including PubMed/MEDLINE, EMBASE, PsycINFO, and the Cochrane Library, ensuring a robust evidence base. The consensus was developed by a panel of six senior experts during a formal consensus meeting held in Madrid. Following a structured discussion and evidence synthesis, a modified Delphi-like process was employed to refine the recommendations. The quality of evidence and the strength of the recommendations were assessed using the grading of recommendations assessment, development, and evaluation (GRADE) approach.

**Results:**

The evidence confirms cariprazine’s efficacy in acute exacerbations, with a distinct advantage in treating predominant negative symptoms and hostility compared to other second-generation antipsychotics (SGAs). Its metabolic-neutral profile and D3-mediated impact on craving also make it a strategic option for patients with dual diagnosis.

**Conclusions:**

Cariprazine is a valuable tool for the stabilization and long-term functional recovery of patients in acute psychiatric units. The consensus provides a practical framework to optimize its clinical application based on high-quality evidence.

## Introduction

Schizophrenia is a chronic and severe mental disorder that alters thinking, perception and behaviour, affecting patient quality of life. Its symptoms are traditionally grouped into 5 domains based on factor analysis: positive, negative, disorganized thinking, hostility/agitation, and anxiety/depression [1]. A key factor in loss of function is cognitive impairment, which compromises attention, memory and executive functions [2].

The pharmacological treatment of schizophrenia is based on antipsychotics, divided into two types: first-generation (first-generation antipsychotics [FGAs] or typical) and second-generation (SGA or atypical) drugs. Both target dopamine D2 receptors to reduce dopaminergic hyperactivity and alleviate positive symptoms [3, 4, 5]. FGAs have been reduced in use because of their extrapyramidal side effects [6].

On the other hand, SGAs are the most commonly used drugs because of their lesser risk of causing extrapyramidal symptoms (EPS). They act on multiple receptors, including D2 receptors, and show strong antagonism with serotonin receptors (5-HT2A and 5-HT1A). These drugs are effective against both positive and some negative symptoms of schizophrenia [6, 7]. However, they can cause adverse metabolic effects, including weight gain, diabetes, and dyslipidaemia [6, 8].

The SGAs include partial agonists (considered third-generation drugs), such as aripiprazole, brexpiprazole and cariprazine, which stabilise dopaminergic activity by partially activating the D2 receptors [5, 9] and thus more adequately regulating functioning of the dopaminergic system in the different brain areas. Cariprazine is notable for having 10-fold higher affinity for D3 receptors compared to D2 receptors [10, 11].

On the other hand, cariprazine is a serotonin 5-HT1A receptor partial agonist and a 5-HT2A and 5-HT2B receptor antagonist. Its action on the D3 receptors and 5-HT receptors is essential for its pro-cognitive and antidepressant effects, and for its superior efficacy in the treatment of primary negative symptoms and in dual disorders with substance use [5, 10, 12].

Other advances include long-acting injectables (LAIs), which improve long-term outcomes and adherence [12, 13], and research into new mechanisms of action to specifically address negative and cognitive symptoms [2, 14, 15]. In this context, LAIs of SGA are particularly useful, and risperidone, paliperidone palmitate and aripiprazole are currently available in formulations of this kind [12]. In addition, clinicians now have access to other formulations, such as olanzapine LAI, as well as various first-generation agents (e.g., haloperidol, zuclopentixol, flupentixol or fluphenazine), providing a more diverse armamentarium to tailor treatment to the individual needs of the patient [16, 17, 18].

The treatment of schizophrenia should also be complemented by psychosocial interventions (e.g., cognitive-behavioural therapy, cognitive remediation, etc.), which are crucial to improve functionality and negative symptoms [19, 20, 21]. Early intervention is key to a better prognosis [22]. Although antipsychotics often allow positive symptoms to be controlled, their efficacy against negative and cognitive symptoms is more limited [23, 24]. Non-adherence, often driven by side effects, increases the risk of relapses and re-hospitalisations [25, 26, 27], making the implementation of interventions more difficult and the prognosis worse. In addition, insufficient implementation of the clinical guidelines in real-world practice causes variability in treatments and affects remission rates and quality of care [21, 28].

Current clinical guidelines for the management of schizophrenia emphasize that stabilization in acute psychiatric care units should not only aim for the reduction of positive symptoms but also lay the groundwork for long-term functional recovery [29]. A critical “hotspot” in current practice is the persistence of negative symptoms and cognitive impairment, which often remain unaddressed during the acute phase and significantly hinder the patient’s reintegration into society [11, 23].

The rationale for this consensus arises from the need to address these shortcomings using evidence-based pharmacological strategies. Cariprazine, a partial agonist with high affinity for D3 receptors, has shown potential in targeting these specific dimensions where traditional D2-centric antipsychotics often fall short [30]. Consequently, this article provides a hierarchical review of its clinical utility, positioning it as a strategic tool for the comprehensive management of the acute patient.

## Materials and Methods

This work was conceived as a narrative review supported by a structured expert consensus. The primary objective was to synthesize current evidence on the clinical utility of cariprazine in the management of acute schizophrenia, and to develop practical recommendations for its use in realworld acute care settings. The review focused on key clinical domains relevant to acute episodes, including positive symptoms, negative symptoms, cognitive impairment, hostility, adherence, and comorbid substance use.

The consensus was developed by a multidisciplinary panel of six senior psychiatrists with extensive experience in the management of severe mental illness in acute care settings:

- Psychiatrists 1: Specialist in refractory schizophrenia and pharmacological innovation. Hospital Universitario Ramón y Cajal [31].
- Psychiatrists 2: Expert in cross-cultural psychiatry and emergency psychiatric care. Hospital Vall d’Hebron/Sant Rafael.
- Psychiatrists 3: Expert in Dual diagnosis (substance use and schizophrenia), providing the evidence-based perspective on comorbid populations [32].
- Psychiatrists 4: Specialist in acute hospitalization protocols and stabilization phases. Hospital Universitario de Navarra.
- Psychiatrists 5: Expert in therapeutic intervention and psychiatric services organization. Hospital Universitario de Badajoz.
- Psychiatrists 6: Senior researcher with expertise in clinical methodology and evidence synthesis, acting as the primary coordinator for the grading of recommendations assessment, development, and evaluation (GRADE) assessment [33]

These six senior psychiatrists, were strategically selected for their extensive experience in acute inpatient units and their dual roles as clinicians and researchers. This group includes internationally recognized experts in pharmacological innovation [31], dual diagnosis [32], and, crucially, clinical research methodology. Dr. Luis Gutiérrez-Rojas and Dr. José Manuel Montes-Rodríguez provided the evidence-based medicine (EBM) expertise required to lead the GRADE assessment and ensure the hierarchical synthesis of the clinical data, thus mitigating the potential bias of a smaller expert group.

### Consensus Development Process

A structured inperson meeting was held in Madrid in February 2025. During this session, the panel defined the scope of the review, the key clinical questions, and the methodological framework for evidence synthesis.

Following the meeting, the experts engaged in an iterative process of literature review, critical appraisal, and manuscript refinement. Although no formal Delphi procedure was conducted, all recommendations were discussed collectively and approved by unanimous agreement. No predefined voting threshold was required, as consensus emerged naturally through discussion and revision.

### Literature Search Strategy

A comprehensive literature search was conducted across four major databases: PubMed/MEDLINE, EMBASE, PsycINFO, and the Cochrane Library [34]. The search covered publications from January 2017 to April 2025 and included articles in English or Spanish.

The search strategy combined controlled vocabulary and freetext terms related to schizophrenia, acute episodes, antipsychotic treatment, negative symptoms, cognitive impairment, and cariprazine. The Boolean structure used across databases was: (schizophrenia OR psychosis) AND (acute OR exacerbation) AND (treatment OR antipsychotic) AND (cariprazine OR “D3 partial agonist”) AND (negative symptoms OR cognition OR hostility OR adherence).

Eligible study types included randomized controlled trials, meta-analyses, systematic reviews, observational studies, clinical guidelines, and expert consensus documents. Editorials, letters, conference abstracts, and duplicate publications were excluded.

### Eligibility Criteria

Studies were included if they met the following criteria:

- Focused on schizophrenia or acute psychotic episodes.
- Reported clinical outcomes relevant to antipsychotic treatment, particularly cariprazine.
- Provided data on symptom domains relevant to acute management (positive, negative, cognitive, hostility/aggression, adherence, or comorbid substance use).
- Employed recognized scientific methodology (randomized placebo-controlled trials (RCTs), systematic reviews, meta-analyses, observational studies, or guidelines).

Studies were excluded if they:

- Lacked original data (e.g., editorials, commentaries).
- Focused exclusively on chronic or stable-phase schizophrenia without relevance to acute management.
- Were not available in English or Spanish.

### Study Selection and Data Extraction

A total of 1450 records were identified across the four databases. After title and abstract screening, 1376 records were excluded for not meeting eligibility criteria. The remaining 74 fulltext articles were assessed for eligibility and included in the final evidence synthesis. No additional records were identified through manual searching.

Data extraction focused on:

- Study design and methodology.
- Patient population.
- Intervention characteristics.
- Clinical outcomes (efficacy, safety, functional measures).
- Relevance to acute schizophrenia management.

### Quality Assessment

The quality and strength of evidence supporting each recommendation were evaluated using the GRADE [35, 36] framework. Evidence was classified as high, moderate, low, or very low, and recommendations were categorized as strong or conditional. When evidence was insufficient or inconsistent, statements were explicitly labeled as expert opinion. The detailed assessment is set out in Table 1 (Ref. [1, 2, 3, 6, 8, 10, 11, 12, 13, 23, 25, 26, 27, 28, 30, 31, 32, 37, 38, 39, 40, 41, 42, 43, 44]).

**Table 1.** **Summary of GRADE assessment**.

Clinical domain	Evidence summary	Limitations	Quality (GRADE)	Strength	Key references (numeric)
Positive symptoms in acute schizophrenia	SGAs, including cariprazine, reduce acute psychotic symptoms; efficacy comparable to other SGAs	short trials; some post-hoc analyses	moderate	strong	[1, 6, 8, 23, 26, 37, 38, 39]
Predominant negative symptoms	pivotal RCT shows superiority of cariprazine vs. risperidone; strong mechanistic rationale via D3 affinity	evidence dominated by one RCT	high	strong	[10, 11, 12, 40]
Cognitive impairment	preclinical evidence suggests pro-cognitive effects; limited clinical data	mostly animal data; indirect human evidence	low	conditional	[10, 26]
Hostility/aggression	post-hoc analyses show reductions in hostility	hostility not a primary endpoint	low	conditional	[2, 41]
Dual disorder (substance use)	partial agonists recommended; D3 modulation may reduce craving	mostly observational evidence	low	conditional	[12, 28, 32, 42]
Metabolic profile	cariprazine shows a metabolically neutral profile	limited long-term data	moderate	strong	[8, 32, 38, 43]
Adherence/partial adherence	long half-life may buffer missed doses	PK-based inference; no adherence RCTs	low	conditional	[3, 25, 27]
FES	SGAs recommended as first-line; cariprazine suitable due to tolerability	no RCTs in FES	low	conditional	[26, 44]
Use in acute care units	effective for acute stabilization; supported by expert experience	no trials specifically in acute units	moderate	strong	[13, 30, 31, 37, 40]

SGAs, second-generation antipsychotics; FES, firstepisode schizophrenia; GRADE, grading of recommendations assessment, development, and evaluation; RCT, randomized placebo-controlled trial; PK, Pharmacokinetics.

The “high” quality rating assigned to the evidence for treating predominant negative symptoms is primarily based on the pivotal study by Németh *et al*. (2017) [40]. While this assessment is dominated by a single trial, the expert panel determined that its rigorous design as a large-scale (n = 461), randomized, double-blind, head-to-head study versus risperidone specifically designed to evaluate this domain provides a direct and robust evidence base. Furthermore, this clinical evidence is reinforced by a strong mechanistic rationale—specifically cariprazine’s unique 10-fold higher affinity for D3 receptors compared to D2 [10, 11]—which the panel concluded justifies a high-quality rating by bridging the gap between pharmacological theory and clinical outcomes, thereby mitigating concerns regarding single-study dependence.

### Preferred Reporting Items for Systematic Review and Meta-Analysis Flow Description

**Fig. 1.**
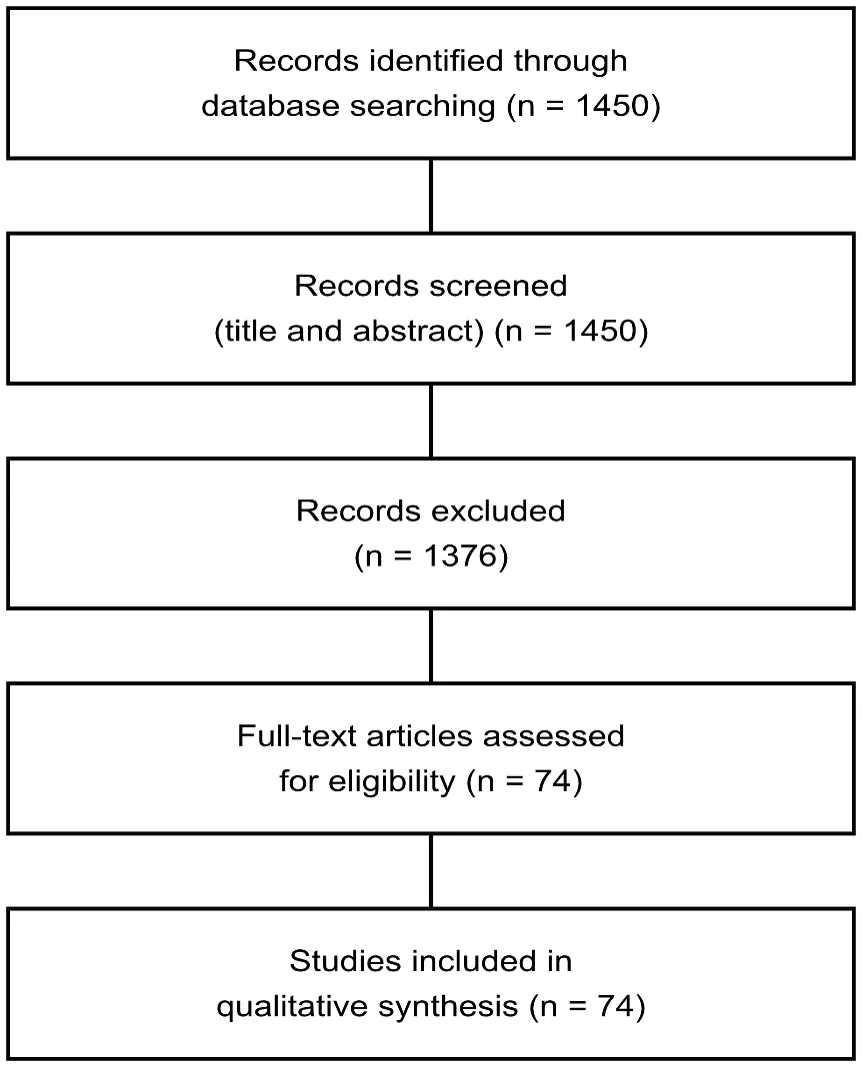
**PRISMA flow diagram of study selection**. PRISMA, Preferred Reporting Items for Systematic Review and Meta-Analysis.

The study selection process followed Preferred Reporting Items for Systematic Review and Meta-Analysis (PRISMA) [45] guidelines (please see Fig. 1).

- Records identified: 1450.
- Records excluded after screening: 1376.
- Fulltext articles assessed: 74.

Studies included in qualitative synthesis: 74.

## Results

### Current Clinical Status

The first contact of patients with schizophrenia with the healthcare system usually occurs in the hospital emergency room, normally due to the presence of positive symptoms (delusions and hallucinations), behavioural changes, psychomotor agitation, and hostility [13, 26, 46]. Relapses are usually due to non-adherence and consequent treatment discontinuation, substance use or the worsening of stressors, as well as in a low social-family support network. In the first crisis, the search for help is often initiated by relatives or primary care physicians [26].

Psychiatric hospitalisation is a common resource for the control of severe symptoms and initial stabilisation. Deciding patient admission is justified by specific clinical criteria, such as the severity of the psychotic symptoms, aggressive behaviour, or physical risk to the patient or others. The containment capacity of the social-family environment to manage the crisis on an outpatient basis is also considered. Although day hospitals allow for stabilising and avoiding admission in many cases, hospital admission may be necessary to prevent the exacerbation of serious conditions, such as decompensation with acute psychotic symptoms in patients with schizophrenia [23].

**Fig. 2.**
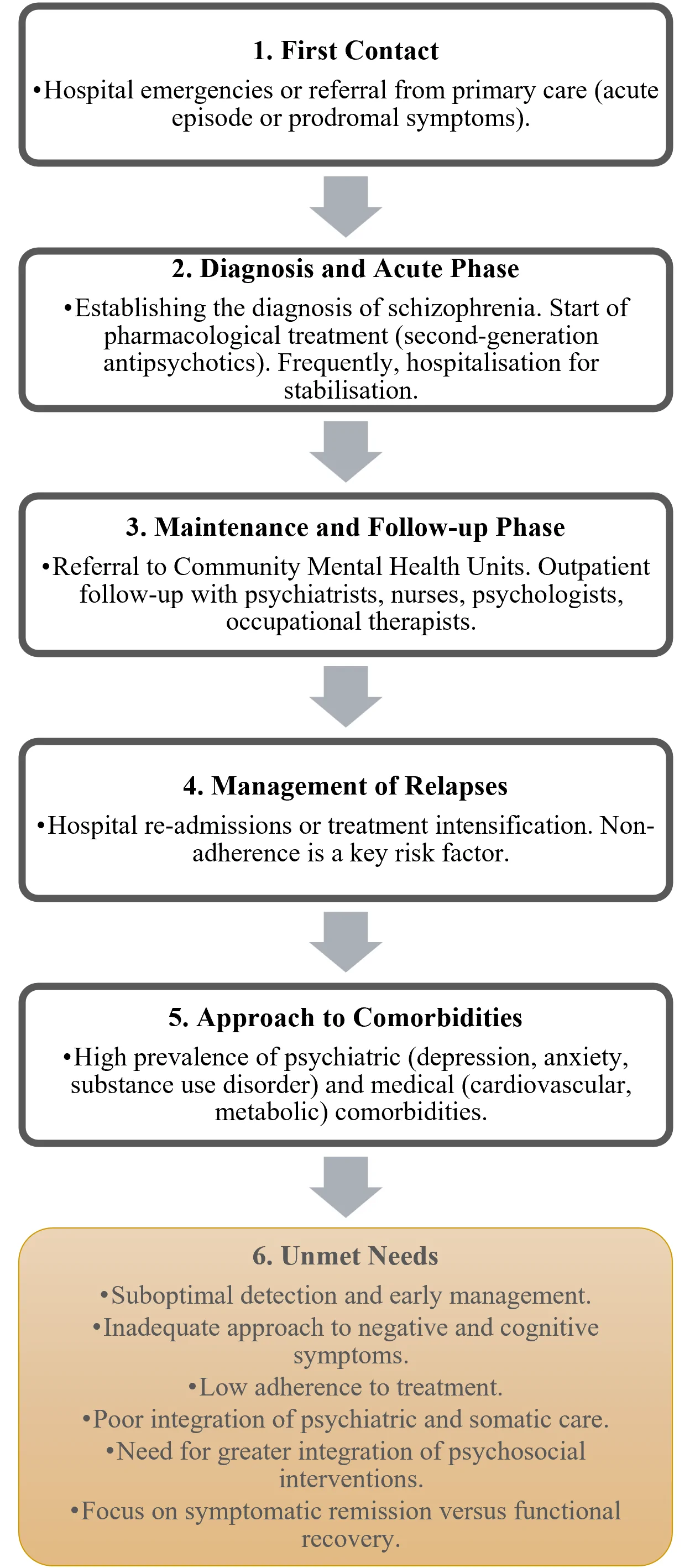
**Flow of patient with acute episode of schizophrenia**.

After discharge (from the emergency room or the short psychiatric hospitalisation unit), a visit to the outpatient mental health unit (MHU) is scheduled within about 7 days. In the maintenance phase, the MHU psychiatrist performs regular monitoring, the frequency of which is adjusted according to the clinical condition and functionality of the patient [13]. These conclusions are summarised in Fig. 2 [47].

Expert panel characterized the profile of patients with schizophrenia most commonly seen in their clinics: individuals 18–35 years of age, living in urban areas, and with family support. They are usually patients with a low socioeconomic level and a high unemployment rate. Clinically, they usually have a family history of schizophrenia. The most common symptom leading to the search for help is disorganised behaviour with serious alterations of the affective-social dimension [26].

A key finding is cognitive deficit (either inherent to the disease or caused by the previous treatments received), since successive episodes of schizophrenia cause cumulative, often irreversible cognitive impairment. In addition, aggressive and hostile behaviour is often associated with substance use, such as cannabis, which has a cumulative negative impact on cognition. Although chronic comorbidities are uncommon in this age range, the use of drugs for other conditions should be considered, particularly for blood glucose control [12, 23, 43].

The panel defined the patient with acute schizophrenia as requiring immediate therapeutic intervention upon the onset or intensification of psychotic symptoms. The therapeutic approach should be comprehensive, addressing not only positive symptoms but also key prognostic factors such as low adherence, social-family support, and impaired patient functionality, cognition, and affectivity.

Main Clinical Characteristics, Course, Impact, and Comorbidities of Schizophrenia are shown in Table 2 (Ref. [11, 23, 28, 42, 43, 46, 48, 49, 50, 51, 52]).

**Table 2.** **Main Clinical Characteristics, Course, Impact and Comorbidities of Schizophrenia**.

Clinical and functional domain	Description
Onset and course	∙ onset: late adolescence or early adulthood [23, 28, 46]
	∙ severe, chronic and recurrent disease [28]
	∙ dramatically interferes with developmental milestones (early onset is associated with high disease burden and disability) [23, 28, 46]
Nuclear dimensions	∙ positive: hallucinations, delusions, and thought disturbances [11, 28]
	∙ negative: avolition, anhedonia, associability, affective flattening, alogia [11]
	∙ cognitive deficit: central characteristic and predictor of daily functioning [43]
Related events	∙ hostility is a symptom domain correlated with aggression. Higher risk if schizophrenia is not treated or there is substance use [28, 48]
	∙ affective-social disorders (common reason for behaviour): depression and anxiety [11, 28]
Socioeconomic status and impact	∙ significant impairment of educational functioning [28, 46]
	∙ low socioeconomic level, may be a consequence of academic and cognitive/functional impairment [49]
	∙ high economic burden on society (50–80% due to indirect costs such as unemployment and early retirement) [50]
Comorbidities	∙ SUDs are a common comorbidity [42, 48]
	∙ very common: cannabis and alcohol use [42, 48]
	∙ higher relapse rates [28, 48]
	∙ poorer adherence [48]
	∙ stressful or hazardous environments as risks factors [28]
	∙ somatic comorbidities: High risk of metabolic syndrome [23, 28, 48]
Risk of suicide	∙ increased due to lack of adherence and exacerbation of symptoms [51, 52]
Other features	∙ highly heritable [42]
	∙ interaction of multiple genes with environmental factors [11, 42]
	∙ increased mortality and reduced life expectancy [28, 46]

SUDs, substance use disorders.

### Treatment Strategies

The treatment of schizophrenia is based on the synergistic combination of antipsychotics and non-pharmacological therapies [19]. Antipsychotics are the mainstay of treatment, and SGAs are recommended as the cornerstone because of their more favourable adverse effects profile compared with FGAs, particularly as regards EPS [23, 38]. In addition, they address both positive and some negative symptoms [8, 53]. Main pharmacological recommendations for schizophrenia management are summarised in Table 3 (Ref. [3, 4, 6, 8, 23, 27, 46, 53]).

**Table 3.** **Presents the key recommendations based on the consulted literature**.

Recommendation	Rationale
Use second generation antipsychotics as first line	improved cognitive profile and lesser risk of EPS [23]
Avoid olanzapine as first line in FES	high burden of metabolic side effects [8]
Clozapine for treatment-resistant schizophrenia	efficacy in treatment-resistant disease, recommended as third-line therapy [53]
Minimise use of anticholinergics and benzodiazepines	negative impact on cognitive functioning [23]
Periodic monitoring and dose optimisation	optimise therapeutic response and minimise side effects [6]
Early intervention in FES	improve long-term outcomes [6, 46]
Consider LAI for non-adherence	effective in maintaining adherence in difficult cases [4, 27]
Partial adherence	the long half-life of cariprazine may offer advantages in maintaining therapeutic levels in partially adherent patients, which could reduce the impact of missed doses [3]

EPS, extrapyramidal symptoms; LAI, long-acting injectable; FES, firstepisode schizophrenia.

The treatment plan should be adapted to each individual patient, taking into account the severity of symptoms, the side effects profile, current comorbidities and patient preferences. Patients with a history of metabolic syndrome may benefit from the use of antipsychotics with a lower risk of weight gain, such as cariprazine, aripiprazole or lurasidone [8, 46, 54].

Patients should be regularly monitored not only for their symptoms of schizophrenia but also for side effects and treatment adherence. Dosing should be adapted according to patient response and tolerability, with the aim of achieving the minimum effective dose [53].

Intervention as early as possible is advised. Treatment should be started with SGAs (due to their efficacy and tolerability) in monotherapy. The dosage will be gradually adjusted to achieve the optimal therapeutic effect while minimising adverse effects [4, 46, 53].

Olanzapine is not recommended for first-line use in firstepisode schizophrenia (FES) due to its adverse metabolic effects, including weight gain and the risk of metabolic syndrome [8, 53]. It is reserved for use in cases of resistance to other treatments [53].

Although the administration of LAI antipsychotics is recommended in another setting, their use in FES is more indicated in patients in whom a high relapse rate is expected, associated with poor adherence [53].

The treatment of negative symptoms in schizophrenia represents a significant challenge and an unmet clinical need [3, 11].

Cognitive impairment is a common feature of schizophrenia, and is associated with poor functional outcomes. While there are no specific drugs approved for the treatment of cognitive impairment in schizophrenia, SGAs with a favourable cognitive profile are recommended, such as cariprazine, aripiprazole and lurasidone. In addition, psychosocial interventions, such as cognitive remediation and physical exercise should be considered as adjunctive therapies [10, 23, 40].

Specifically, cariprazine has been postulated to have a direct pro-cognitive effect due to its greater affinity for D3 receptors. In animal models, cariprazine has shown pro-cognitive activity, reversing deficits in working memory, attention switching and recognition memory [10]. (A quantitative synthesis of cariprazine efficacy by symptom domains can be found in Table 4, Ref. [1, 10, 40, 41, 55, 56, 57]).

**Table 4.** **Quantitative synthesis of cariprazine efficacy by symptom domains**.

Domain hierarchy	Clinical domain	Key evidence/study design	Quantitative results (effect size/*p*-value)
Nuclear dimensions	negative symptoms	randomized, double-blind, phase IIIb head-to-head study vs. risperidone (n = 461)	LSMD: –1.46; *p* = 0.002 in the PANSS-PSNS score at week 26 [40]
	positive symptoms	meta-analysis of randomized controlled trials on efficacy and safety	confirmation of superior efficacy in total symptom reduction (PANSS total) with a favorable tolerability profile [55]
	cognitive symptoms	post-hoc analysis of the PANSS cognitive factor in acutely exacerbated patients	significantly greater improvement in the cognitive factor vs. risperidone (LSMD –0.73; *p* = 0.02) and vs. placebo [10]
Related events	hostility	pooled post-hoc analysis of 3 RCTs in acute schizophrenia	significant reduction in PANSS hostility score vs. placebo (*p * < 0.05); superior efficacy demonstrated compared to aripiprazole [41, 56, 57]
	depressive symptoms	RCT evaluating the MADRS scale	statistically significant improvements in MADRS total score vs. placebo from week 2 onwards (*p * < 0.05) [1]

PANSS, Positive and Negative Syndrome Scale; PANSS-PSNS, positive scale-negative scale; MADRS, Montgomery-Åsberg Depression Rating Scale; LSMD, least squares mean difference; RCT, randomized placebo-controlled trial.

To minimise cognitive side effects, it is advisable to minimise drugs with a high cholinergic burden, since they may worsen cognitive impairment. In addition, the use of benzodiazepines is recommended to be moderate, as they can also impair cognitive function [23].

In patients with comorbid depression, the literature recommends the use of antipsychotics with mood stabilising properties, such as quetiapine or the combination of olanzapine and fluoxetine, together with adjunctive antidepressants if necessary [58, 59].

Adults and young people with schizophrenia and regular substance use or a concomitant substance use disorder (SUD; dual diagnosis) are among the most vulnerable groups, with a series of clinical, social and biological factors that place them at high risk. These include an increased risk of relapses and hospitalisations, as well as of symptom exacerbations with an increased risk of mortality and suicide, as well as overall poorer functioning [12, 48, 60]. These patients pose a challenge to healthcare systems, among other aspects, due to their greater medication adherence problems and poorer outcomes [12]. For the management of schizophrenia in patients with dual diagnosis, partial agonists (cariprazine, aripiprazole) are recommended as first-line treatment [7, 38], in addition to concomitant treatment of the addiction.

Women with schizophrenia require an individualised therapeutic approach due to their differences in metabolism and tolerance to antipsychotics. Dose adjustment and monitoring are advised, hormone therapies such as raloxifene in postmenopausal women should be considered, and antipsychotic drugs should be used with caution during pregnancy and lactation, avoiding clozapine because of its potential risk [58]. Psychosocial interventions are an essential component of integral schizophrenia treatment, improving the course of the disease and the long-term outcomes [26]. They help patients manage everyday challenges and symptoms while pursuing their personal goals, such as education, employment and establishing meaningful relationships. Early implementation is critical in order to prevent cumulative disability [26].

Cognitive-behavioural therapy (CBT) is recommended as first-line treatment by guidelines such as National Institute for Health and Clinical Excellence (NICE) and the Royal Australian and New Zealand College of Psychiatrists (RANZCP) [19]. This therapy has been shown to improve functional outcomes in the real world, including occupational and social functioning. In addition, it has been shown to be effective in reducing positive and negative symptoms, as well as the severity of general and total psychopathology [19, 23].

Cognitive remediation (CR) is the psychosocial intervention with the greatest evidence of efficacy in addressing cognitive impairment in schizophrenia [23]. Family interventions and psychoeducation are also crucial, as they improve patient and family functioning and the course of the disease [26, 46].

Other interventions have also been investigated, such as social skills training, supported employment and assertive community treatment, each of which has shown benefits in specific areas (sociability and employment) [19, 23, 26].

### Core Challenges

Despite advances in pharmacology and treatment guidelines, expert consensus has identified ongoing challenges and unmet clinical needs that preclude optimising the prognosis of patients with schizophrenia.

Non-adherence to treatment is highlighted as one of the main causes of relapses and re-hospitalisations, with devastating negative consequences for the prognosis, including increased psychotic symptoms, cognitive impairment, and greater healthcare costs, as well as a progressive loss of functionality and capacity for social reintegration [25].

Factors influencing non-adherence include personal aspects, particularly lack of awareness of the disease, which is a critical risk factor for non-adherence. A negative attitude towards the medication and concerns about its necessity contribute to the problem [25, 27]. In addition, perceived adverse effects of psychotropic drugs that affect daily activities or quality of life also have an influence on adherence. Agitation, EPS, sedation, cognitive and hormonal alterations, and metabolic effects are of particular concern to patients [6].

Forgetfulness caused by the cognitive impairment inherent to the disease also negatively influences adherence, making it a complex and multifactorial problem [25, 26].

Coping skills and the establishment of routines—factors highly influenced by the functional and psychosocial functioning of the patient—play a key role in adherence [25, 26].

In addition, there are other factors that determine non-adherence, such as substance use, family and social support, or accessibility to services that make the problem of adherence in patients with schizophrenia a complex and multifactorial problem [25]. Addressing non-adherence requires identification of the specific reasons and unique needs of each patient in order to design individualised intervention strategies [25, 26].

Dual diagnosis (DD), refers to the comorbid condition of the presence of at least one SUD and any other mental illness, including the psychotic and affective categories. The World Health Organisation (WHO) definition for DD specifies the “co-existence in the same individual of a SUD and another psychiatric disorder” [12]. This comorbidity is markedly high in schizophrenia; almost half of all individuals diagnosed with schizophrenia also have SUD [12]. The literature shows that 48% of people with schizophrenia develop a substance addiction at some point in their lives [12]. Cannabis is often the substance of abuse, a finding that confirms that this molecule is the most commonly used worldwide and harms mental health [12].

The presence of dual diagnosis has a considerable clinical and healthcare impact, and is often underdiagnosed and insufficiently treated, which makes it complex to manage. As compared to mental disease or SUD alone, DD is associated with a delayed diagnosis, worsening of the prognosis, and greater complications, such as the exacerbation of psychiatric symptoms, a greater frequency of hospital admissions, and poorer medication adherence [12].

In addition, dual diagnosis is associated with an increase in aggressive and violent behaviour. The relationship between the two conditions is complex, suggesting bidirectional models where mental illness may trigger substance abuse, or vice versa, and both conditions may interact and exacerbate each other. Genetic analyses also support the existence of an underlying shared biology driving this overlap of substance dependence and schizophrenia [12, 42].

A crucial challenge identified is that the implementation of clinical guidelines in the daily practice of acute psychiatric hospitalisation units is not widespread [13]. This inconsistency in adherence to the guides has direct repercussions on remission rates and quality of care [61]. The problem does not lie in the lack of knowledge about best practices (since there is wide international consensus on the use of SGAs and clozapine), but in the difficulties for their effective application in the real world—including lack of knowledge of the guidelines on the part of clinicians or limited access to monitoring of specific drugs such as clozapine [62].

### Treatment Optimization and Future Directions

In the opinion of the authors, the recommendations on the treatment of schizophrenia, both those contained in the published guidelines and those derived from real-world data (RWD) studies, suffer from an exclusive focus on patient clinical criteria. As schizophrenia is a complex disease, the authors consider that there are a number of other criteria that should be taken into account when making intervention decisions in people with acute psychotic decompensations of schizophrenia [6, 12, 63]. The consensus proposes an update on the management of these acute decompensations of schizophrenia, emphasising a comprehensive, individualised approach based on symptomatic domains, in order to optimise the patient prognosis and functionality from the early stages.

Individualised and comprehensive approach: the treatment plan should be adapted to each individual patient, considering the severity of symptoms, the side effects profile, current comorbidities, and patient preferences. A holistic approach should be adopted that addresses, beyond positive symptoms, low adherence, social-family support, functional impairment, cognition, and affectivity [46, 54].

Early intervention: intervention should be as early as possible. This is crucial for recovery and for improving the long-term outcomes, as early and aggressive intervention can alter the trajectory of the disease [23, 26].

Close monitoring: Close longitudinal follow-up is mandatory to avoid symptoms exacerbation, prevent relapse, and maintain stable antipsychotic therapy. Follow-up should be comprehensive, including monitoring of symptoms of schizophrenia, side effects, and adherence, in order to achieve the lowest effective dose. In the FES, recommended follow-up ranges from 2 to 5 years [23, 26].

SGAs (amisulpride, quetiapine, risperidone, olanzapine, aripiprazole and ziprasidone) are recommended as first-line pharmacological treatment, especially in the early stages or in the FES [29]. Partial agonists (cariprazine, aripiprazole, brexpiprazole), on the other hand, are effective in controlling positive symptoms and have shown benefits for patients with comorbidity due to SUDs [2, 6, 12]. Specifically, cariprazine has demonstrated superior efficacy over placebo in the control of positive and negative symptoms. It has also shown comparable efficacy to risperidone or aripiprazole in reducing positive symptoms [3, 10, 64]. This improvement in negative symptoms is associated with its greater affinity for D3 receptors, and a large controlled clinical trial demonstrated that this drug may be effective in controlling predominant negative symptoms in patients with chronic schizophrenia [10, 40].

In the case of refractory schizophrenia, clozapine universally remains the medication of choice, and should be started as soon as possible in patients who fail to respond to two or more previous antipsychotics [65].

In cases where patients show prolonged resistance to drug treatments, electroconvulsive therapy (ECT) has become a crucial option [23]. Specifically, it has been investigated as an augmentation to clozapine (ECT in combination with clozapine) [53]. In addition, ECT has been shown to help reduce negative symptoms in these patients [7].

In clozapine-resistant patients, ECT has been shown to improve positive symptoms and overall clinical outcomes. The literature suggests that ECT may be an effective strategy (particularly in refractory cases, with catatonic-like symptoms, or with a greater incidence of affective clinical symptoms), particularly when combined with clozapine, because it results in significant clinical improvements in most patients [23, 53].

The treatment of primary negative symptoms remains a great challenge and an unmet clinical need. Current therapies provide limited improvements and lack standardisation. These symptoms are associated with poorer functional outcomes, less remission and poorer quality of life [61].

Compared with FGAs, some SGAs such as cariprazine and amisulpride have demonstrated greater efficacy in this domain [66]. This can be attributed to their different pharmacological profiles, which include lower affinity for the dopamine D2 receptor and action on other neurotransmitter systems. Specifically, cariprazine has demonstrated superior efficacy in the treatment of primary negative symptoms such as avolition and anhedonia, and is considered a treatment of choice for these symptoms [67].

In this regard, in a randomised, double-blind, phase IIIb study conducted in 11 countries involving 461 patients with schizophrenia in which changes in the positive scale-negative scale (PANSS-PSNS) score were measured, cariprazine was shown to be more effective than risperidone in improving predominant negative symptoms in patients with schizophrenia [40].

After the literature review, the authors of the work concluded that cariprazine demonstrated superior efficacy in the treatment of predominant negative symptoms compared to another second-generation antipsychotic. In this regard, they suggest that patients who have achieved adequate control of positive symptoms but persist with debilitating negative symptoms under another antipsychotic treatment may derive significant clinical benefit when switching treatment to cariprazine [40].

SGAs such as cariprazine and amisulpride are preferred over FGAs due to their better functional outcomes and lesser cognitive impairment. These drugs can help control negative and affective symptoms by acting upon multiple neurotransmitter systems, including dopamine and serotonin receptors [6, 66].

The broader efficacy of cariprazine in the treatment of affective symptoms may be attributed to its ability to modulate dopamine and serotonin receptors, which are key targets in the treatment of mood disorders [38, 54].

Although cognitive impairment is a central feature of schizophrenia, current drugs show little efficacy, and greater relevance is thus given to non-pharmacological approaches [26, 43, 46].

SGAs with a favourable cognitive profile are recommended, such as cariprazine, aripiprazole and lurasidone, which have a different mechanism of action than traditional antipsychotics, and have shown a more positive impact on cognitive function [28].

Evidence from a post-hoc analysis of a Phase III study suggests that cariprazine significantly improves cognitive symptoms on the Positive and Negative Syndrome Scale (PANSS) cognitive factor score. When compared with risperidone, cariprazine demonstrated greater improvement in cognitive symptoms, especially in acutely exacerbated patients [3, 10]. Investigators believe that these procognitive effects may be due to partial D3 receptor agonism, which can enhance dopaminergic neurotransmission in cortical areas of the brain [3, 10].

Anxiety and mood disorders are prominent and often precede psychotic relapses [1]. In this context, FGAs (e.g., haloperidol) are used for rapid control of acute agitation, but carry an increased risk of EPS [29].

Clozapine and intramuscular formulations of some SGAs (olanzapine and ziprasidone, whose effect only lasts for a few hours), including LAIs, may be effective in controlling agitation and aggression in acute situations, generally with a lower risk of EPS than haloperidol [29].

Cariprazine has shown significant efficacy in reducing hostility in post-hoc analyses from 3 randomized controlled trials—an effect related to its partial D3 and D2 agonism [2, 55]. Real-world studies in patients with acute exacerbation of schizophrenia treated with cariprazine have demonstrated a significant reduction in hostility scores, and also in positive and negative symptoms [2]

Anxiety and depression are common in schizophrenia, aggravate functional impairment and quality of life, increase suicidal risk, and their therapeutic management is complicated [29, 55].

The clinical management of schizophrenia is complicated by a significant overlap between the negative and depressive domains, which share core features such as anhedonia, emotional flatness, anergia and abulia/avolition [1]. The addition of antidepressants (particularly mirtazapine and fluoxetine) has become an established standard treatment strategy, with various meta-analyses demonstrating that they are moderately effective in alleviating the burden of negative symptoms compared with antipsychotic monotherapy [7].

Although the use of antidepressants is considered effective in the treatment of negative symptoms in general, their efficacy remains a subject of considerable controversy. In this setting, a meta-analysis conducted in 2025 has suggested that the adjunctive use of mirtazapine and duloxetine may effectively improve the negative symptoms of schizophrenia in patients receiving stable antipsychotic treatment [59].

SGAs are preferred over FGAs because of their better functional outcomes and lesser cognitive decline. Quetiapine, lurasidone and aripiprazole have shown efficacy in reducing depressive symptoms [5, 6, 23, 63].

Another promising atypical antipsychotic is cariprazine, which in a randomised placebo-controlled trial showed significant improvements in depressive symptoms as measured by the Montgomery-Åsberg Depression Rating Scale (MADRS). Patients who received cariprazine showed greater improvements in the severity of depressive symptoms compared with patients who received placebo [1]. Another study confirmed the efficacy of cariprazine as adjunctive therapy, with significant improvements in MADRS scores observed as early as week two and maintained through week six. The 1.5 mg/day dose was particularly effective, showing a statistically significant difference from placebo in reducing depressive symptoms [1]. A summary of recommended pharmacological treatments for schizophrenia by symptoms domain can be found in Table 5 (Ref. [1, 3, 4, 6, 7, 10, 11, 23, 29, 37, 40, 46, 53, 59, 61, 63]).

**Table 5.** **Recommended pharmacological treatments for schizophrenia by symptoms domain**.

Hierarchy of symptoms	Symptoms domain	Recommended treatments	Comments
Nuclear dimensions	positive symptoms	First line: SGAs (atypical): risperidone, olanzapine, aripiprazole, paliperidone, lurasidone, cariprazine [4, 46, 63].	better extrapyramidal side effects profile [3, 6, 63].
		First-generation antipsychotics (FGAs) such as chlorpromazine, fluphenazine, etc. They show similar efficacy, but with a higher incidence of EPS [3, 53]. Haloperidol for acute agitation [1, 63].	
		Clozapine: Reserved for treatment-resistant schizophrenia (failure to ≥2 antipsychotics with adequate doses and time). It is the most effective option for resistant positive symptoms [53].	Clozapine requires strict haematological monitoring. The goal is the lowest effective dose to control symptoms and minimise side effects [53].
	negative symptoms	It is postulated that some SGAs may have a slightly greater effect on negative symptoms than FGAs, although the evidence is limited and the effects are modest [11, 61, 63]. Cariprazine has shown greater efficacy in reducing negative symptoms than risperidone and aripiprazole [1, 11].	Negative symptoms are difficult to treat pharmacologically [3, 11, 46]. It is crucial to differentiate primary negative symptoms from secondary negative symptoms [1, 11, 61].
	cognitive symptoms	Some SGAs: Lurasidone, cariprazine and other SGAs may have a slightly better or less detrimental cognitive profile than FGAs, but the improvements are at best very modest [23, 46].	
		Cariprazine has shown procognitive effects independent of the improvement of other symptoms [1, 10].	
Related events	aggression/ hostility	Acute management: Intramuscular antipsychotics (SGAs such as olanzapine, ziprasidone; FGAs such as haloperidol), often in combination with benzodiazepines (lorazepam IM) [1, 63].	
		Clozapine: Considered the treatment of choice for persistent and severe aggression, especially in refractory patients [29, 63].	
		Adequate doses of the primary antipsychotic (SGA or FGA) [1, 37].	
		Cariprazine has shown significant reductions in hostility compared to aripiprazole [40].	
	anxiety/ depression	Antidepressants (SSRI/SNRI): sertraline, escitalopram, venlafaxine etc. They are the treatment of choice for significant comorbid depressive symptoms, added to the antipsychotic [59]. Some SGAs with antidepressant/anxiolytic effect: Quetiapine: Often used for its sedative and anxiolytic properties [63]. Lurasidone: Has an indication for bipolar depression and has shown effects on depressive symptoms in schizophrenia [3, 6]. Olanzapine: Approved for bipolar depression [1]. Aripiprazole/brexpiprazole: Used as adjuvants in major depression [3, 63]. Cariprazine shows anxiolytic and antidepressant effects superior to aripiprazole. Indication in bipolar depression [1, 3].	Depression and anxiety are very common in schizophrenia. It is important to treat these symptoms, as they impact functioning and risk of suicide. Some antipsychotics may be intrinsically helpful, but an additional antidepressant is often required [1, 3, 7, 63].

SGAs, second-generation antipsychotics; EPS, extrapyramidal symptoms; SSRI, selective serotonine reuptake inhibitor; SNRI, serotonine noradrenaline reuptake inhibitor; IM, intramuscular.

The treatment of schizophrenia differs significantly between the FES and subsequent episodes, mainly due to the different clinical and therapeutic needs at each stage. The FES represents a critical period for intervention, as early and aggressive treatment can significantly alter the disease trajectory. Studies suggest that early intervention may improve remission and recovery rates compared to later stages, although the need to investigate and optimise treatment strategies persists because the remission rates remain low [51, 68, 69].

In contrast, the management of subsequent episodes focuses on maintenance, prevention of relapse, and the treatment of resistance. As relapses are common, continued antipsychotic treatment is recommended to maintain symptoms remission [70, 71]. In any case, for the management of subsequent episodes and functional recovery, it is essential to continue emphasising non-pharmacological interventions, such as CBT and family support, as they help address the broader psychosocial aspects of the disorder [10, 26].

Non-adherence has devastating negative consequences for the patient’s prognosis, including an increased risk of relapses and hospitalisations, increased psychotic symptoms and longer periods of remission, higher suicide rates, greater cognitive impairment, poorer quality of life, and a direct impact on families and caregivers. In addition, it is associated with a marked increase in the use of healthcare services and increases the costs associated with the treatment of schizophrenia [25].

Coping skills and the establishment of routines—factors highly influenced by the functional and psychosocial functioning of the patient—play a key role in adherence [25, 26].

LAIs for adherence: LAIs represent a key strategy to improve adherence and prevent relapse, offering the highest relapse prevention rates. The risk of re-hospitalisation is significantly lower with LAI monotherapy than with oral antipsychotics or no treatment [26, 72].

Education and simplification: To improve adherence, adequate patient and family education about the disease is required, together with simplification of the treatment regimens [73].

Continuous monitoring: Clinicians should be aware of the need for early intervention with individualised treatment plans and continuous monitoring of symptoms and side effects [23, 26, 74].

The literature recommends the use of partial agonists (cariprazine, aripiprazole) as first-line treatment for schizophrenia with SUD. Cariprazine has received particular attention due to its affinity for D3 receptors, making it useful for managing cognition and substance use, as supported by observational studies in patients with cannabis use disorder and real-world evidence from clinical practice [3, 10, 12, 48].

Neuromodulation techniques, particularly repetitive transcranial magnetic stimulation (rTMS), have shown promise in reducing craving and use of substances in patients with schizophrenia, but more research is needed to optimise these interventions, and always in combination with drug treatment [44]. A summary of the practical implementation of strategies for the Spanish National Health System (SNS) in the management of schizophrenia, can be found in Table 6 (Ref. [3, 27, 62, 74]).

**Table 6.** **Practical implementation strategies for the SNS**.

Barrier category	Identified challenge	Proposed clinical strategy
Adherence/clinical	High relapse rates due to non-adherence and lack of disease awareness.	Early LAI transition: Recommend Long-Acting Injectables early in the course of the disease and simplify oral regimens (e.g., cariprazine’s long half-life) to mitigate the impact of missed doses [3, 27].
Resource constraints	Limited specialized staff for long-term psychotherapy.	Family-centered psychoeducation: Implement structured family interventions, which have shown high efficacy in reducing relapses even in resource-constrained environments [74].
System continuity	Fragmented follow-up between acute psychiatric care units and MHUs.	Shared-care models: Establish “warm handoff” protocols where the first outpatient visit is scheduled within 7 days of discharge and involve primary care GPs in metabolic monitoring [62].

LAI, long-acting injectable; MHUs, outpatient mental health units; SNS, spanish national health system; GPs, general practitioners.

## Discussion

The present review, resulting from a consensus process among expert psychiatrists in Spain, provides a comprehensive and pragmatic characterisation of the current management of schizophrenia. This methodological approach, integrating literature review with clinical experience, is crucial in an area where the application of guidelines is not always widely used. Therefore, the consensus represents a conscious effort to close the gap between scientific evidence and its practical implementation in the Spanish clinical setting.

When interpreting these findings, it is important to distinguish between primary trial data and post-hoc analyses (such as those for hostility and cognition), which should be considered hypothesis-generating. Furthermore, while real-world evidence provides high external validity for the Spanish setting, these studies lack the experimental control of RCTs.

Detailed characterisation of the flow of the patient, which begins in the emergency room and continues with a high relapse rate, underscores the need for a paradigm shift in treatment. Consensus defines adequate intervention as one that transcends the mere remission of positive symptoms. Treatment success should be measured through a holistic and functional view, proactively addressing crucial factors such as low adherence, cognitive and functional impairment, affectivity and social-family support.

This approach is critical because significant challenges persist in key domains for long-term functionality, particularly negative symptoms and cognitive impairment. Non-adherence remains a central challenge, driven by side effects and lack of awareness of the disease. Table 7 offers a clear summary of the consensus findings and the proposals of this review.

**Table 7.** **Summary of key findings of the consensus first meeting and proposals of the current review**.

Consensus/proposal area	Key findings of consensus/details of proposal	Clinical relevance/implication
Patient flow	First contact in the emergency room; relapses due to non-adherence and substance use; follow-up in MHU for stability and functionality.	Guide for therapeutic intervention and optimisation of resources in the Spanish context, identifying critical points of intervention.
Profile of the acute patient	Young people (18–35 years), stable urban/family environment, frequent unemployment, cumulative cognitive deficits, history of psychosis, disorganised behaviour.	Identifies vulnerable subgroups and the need for an early cognitive approach to improve the long-term functional prognosis.
Hospital admission criteria	Severity of symptoms, aggression, physical risk to self/others, family incapacity for outpatient management; key role of day hospitals.	Provides clear criteria for making admission decisions, promoting the efficient use of resources and healthcare alternatives.
Disease awareness and education	Key aspect for adequate management; need for education of patients and relatives.	Highlights the importance of psycho-education as a cornerstone for adherence and family support, which are key to the prognosis.
Treatment approach by symptoms domains	Therapeutic intervention targeted to positive, negative, cognitive and affective symptoms, aggression, and factors such as adherence and social-family support.	Promotes comprehensive and personalised treatment, recognising the complexity of the disease beyond positive symptoms, to improve long-term prognosis and quality of life.
Adherence strategies	Minimising side effects, patient education, simplifying regimens, improving access to care.	Addresses one of the main barriers to successful treatment, proposing practical solutions focused on tolerability and accessibility.
Family support	Essential for reducing relapses and improving adherence; family psycho-education as key strategy.	Highlights the central role of the family as co-therapist and the need for interventions that strengthen their support capacity.

MHU, outpatient mental health unit.

The recommendations of the current review are fully in line with the main international guides for management of the disease. Organisations such as the American Psychiatric Association (APA 2020) [29], the British Association of Psychopharmacology (BAP 2020) [75], the Canadian Psychiatric Association (CPA 2017) [76] and RANZCP (RANZCP 2016) [77] converge on promoting a multifaceted approach [29]. Table 8 (Ref. [3, 6, 10, 12, 23, 24, 29, 30, 31, 32, 40, 48, 64, 73]) shows a comparison of the current consensus vs. international clinical guidelines.

**Table 8.** **Comparison of the current consensus vs. international clinical guidelines**.

Recommendation area	International guidelines (APA, BAP, CPA, RANZCP)	Current consensus (cariprazine focus)	Alignment innovation (added value)
First-line treatment	Unanimous recommendation of SGAs [29].	Aligned. Reinforces SGAs as the cornerstone, prioritizing side-effect profiles from the onset.	Full alignment. Focuses on personalization based on the acute patient profile [31].
Negative symptoms	Identified as an unmet clinical need; limited evidence for most SGAs [24].	Innovation. Positions cariprazine as the treatment of choice based on high-quality head-to-head evidence vs. risperidone [40].	New information. Provides specific efficacy data (LSMD –1.46) not detailed in older guidelines [40].
Cognitive impairment	General recommendation to use drugs with lower anticholinergic burden [23].	Specificity. Proposes cariprazine due to its pro-cognitive effect mediated by D3 receptors, supported by post-hoc analysis [10].	Innovation. Links the D3 mechanism with functional outcomes in the acute phase [10].
Dual diagnosis (SUD)	General recommendations for integrated management [32].	Pragmatism. Recommends partial agonists (cariprazine, aripiprazole) as specific first-line treatment for these patients [12, 32].	New information. Integrates recent real-world observational evidence on cannabis use disorder management [48].
Hostility aggression	Clozapine for persistent aggression; IM SGAs for acute agitation [6]	Aligned & expanded. Maintains clozapine use but highlights hostility reduction with oral cariprazine in acute patients [30, 64]	Nuanced alignment. Offers an effective oral alternative for managing hostility outside of immediate emergencies [30, 64]
Therapeutic adherence	Emphasis on psychoeducation and use of LAIs [27]	Strategic. Combines early LAI use with cariprazine’s long half-life to mitigate the impact of missed doses [3]	New information. Proposes treatment regimen simplification as a critical implementation strategy [73]

SGAs, second-generation antipsychotics; LAI, long-acting injectables; SUD, substance use disorder; LSMD, least squares mean difference; APA, the American Psychiatric Association; BAP, the British Association of Psychopharmacology; CPA, the Canadian Psychiatric Association; RANZCP, the Royal Australian and New Zealand College of Psychiatrists; IM, intramuscular.

Despite advances in the management and development of treatments, there are still unmet needs in aspects such as negative symptom control, cognitive impairment and dual pathologies, among others [10, 12, 23, 46].

In this context of unmet needs, cariprazine emerges as a promising treatment option with a broad efficacy profile across multiple symptoms domains [73]: it has demonstrated superior efficacy to placebo and risperidone in improving predominant negative symptoms in schizophrenia [48]. Its unique mechanism of action, characterized by D2/D3 partial agonism and a high affinity for D3 receptors, provides the neurobiological basis for its efficacy in areas historically resistant to treatment [48]. Specifically, controlled studies have shown that cariprazine is more effective than risperidone in improving predominant negative symptoms, positioning it as a treatment of choice in this domain. This finding is crucial, as it represents a significant advance in an area considered intractable [40].

In addition to its impact on negative symptoms, the evidence suggests that cariprazine improves cognitive symptoms, affective symptoms (anxiety and depression) and is advantageous for the management of dual diagnosis (schizophrenia and SUD), where partial agonists are the recommended first line [12, 32].

To counteract non-adherence, LAIs represent a key strategy, offering the highest relapse and re-hospitalisation prevention rates compared to oral formulations [13, 28]. The implementation of LAIs, along with family psychoeducation and simplification of treatment regimens, is essential to ensure long-term stability and improve overall functionality [72].

The persistence of the gap between evidence and practice, and the urgent need to manage physical comorbidities, makes it necessary to focus future research on the science of implementation and development of integrated care models for the holistic management of physical and mental health.

Ultimately, this work provides a practical roadmap for improving the quality of life and long-term prognosis of patients, highlighting the potential of cariprazine and the need to prioritise functionality, adherence and psychosocial support from the early stages.

### Limitations

This review has several limitations that should be considered when interpreting the findings. First, the consensus was developed by a panel of six experts, which, while providing specialized clinical authority, may limit the generalizability of the practical recommendations to other healthcare settings outside of Spain.

Second, a methodological limitation of the literature search must be acknowledged. The Boolean search string utilized was specifically centered on cariprazine-related terms and its pharmacological mechanism (e.g., “D3 partial agonist”). While this focused approach was designed to ensure a thorough synthesis of cariprazine’s specific clinical profile, it may have limited the retrieval of broader comparative literature concerning other SGAs. Therefore, the search should be viewed as a targeted evidence synthesis rather than a fully comprehensive systematic review of the entire SGA class.

Furthermore, regarding the evidence for cariprazine’s clinical utility, several findings related to hostility and cognitive symptoms are derived from post-hoc analyses or observational/real-world studies rather than primary RCT endpoints. These exploratory data should be considered hypothesis-generating and interpreted with caution. Additionally, many of the cited trials were of relatively short duration, which may not fully reflect long-term maintenance outcomes. Finally, it is important to disclose that the consensus meetings and communication were facilitated with practical support from Recordati Spain, although the authors maintain full responsibility for the content and conclusions of this review.

## Conclusions

The management of schizophrenia in acute psychiatric care units should abandon the generic approach in favour of a therapeutic hierarchy based on specific clinical profiles. The use of SGAs in monotherapy as first-line treatment is established as an essential measure, prioritising low doses in the First-episode psychosis (FEP) to preserve long-term functionality. Among the recommended strategies, cariprazine is positioned as the treatment of choice for patients with predominant negative symptoms (GRADE evidence: High), supported by its superiority over risperidone in randomised clinical trials. To ensure adherence and prevent the “revolving door” effect in the Spanish setting, early transition to LAIs or the use of long half-life molecules such as cariprazine is recommended, especially in cases of dual diagnosis (GRADE evidence: moderate). Finally, it is optional but necessary to integrate psychosocial interventions (such as family education and cognitive remediation) and decision algorithms that include preferential referral to day hospitals to close the gap between the stabilisation of positive symptoms and complete functional recovery. Cariprazine emerges as a promising option, with its efficacy in negative symptoms supported by a head-to-head RCT and its benefits in cognition and hostility further suggested by post-hoc analysis.

## Availability of Data and Materials

Not applicable.
